# Quantitative proteomics analysis of zebrafish exposed to sub-lethal dosages of β-methyl-amino-L-alanine (BMAA)

**DOI:** 10.1038/srep29631

**Published:** 2016-07-12

**Authors:** Ann Kristin Frøyset, Essa Ahsan Khan, Kari Espolin Fladmark

**Affiliations:** 1 Department of Molecular Biology, University of Bergen, Thormøhlensgate 55, 5020 Bergen, Norway

## Abstract

The non-protein amino acid β-methylamino-L-alanine (BMAA) is a neurotoxin present in microalgae and shown to accumulate in the food web. BMAA has been linked to the complex neurodegenerative disorder of Guam and to increased incidents sporadic ALS. Two main neurotoxic routes are suggested; an excitotoxic by acting as an agonist towards glutamate receptors and a metabolic by misincorporating into cellular proteins. We have used zebrafish, an increasingly used model for neurodegenerative diseases, to further identify signaling components involved in BMAA-induced toxicity. Zebrafish embryos were exposed to sub-lethal dosages of BMAA and a label-free proteomics analysis was conducted on larvae 4 days post fertilization. The exposed larvae showed no developmental abnormalities, but a reduced heart rate and increased expression of GSK3 isoforms. Search towards a reviewed database containing 2968 entries identified 480 proteins. Only 17 of these were regulated 2-fold or more in the exposed larvae. Seven of these proteins could be associated to glutamate receptor signaling and recycling. The remaining nine have all been linked to disturbance in protein homeostasis, reactive oxygen species (ROS) development or neuronal cell death. We also found that BMAA influenced the endocannabinoid system by up-regulation of fatty acid amide hydrolase (FAAH) and that FAAH inhibitor URB597 reduced the BMAA effect on heart rate and GSK3 expression.

β-methylamino-L-alanine (BMAA), a neurotoxin and non-protein amino acid produced by wide range of cyanobacteria^1^ and diatoms^2^, has been suggested to be a potential environmental factor in the development of neurodegenerative diseases^3^,^4^. This association was originally proposed from observations on the island of Guam in the Western Pacific Ocean. Here, high incidence of parkinsonism-dementia complex (PDC) together with amyotrophic lateral sclerosis (ALS) was found^5^. Post-mortem brain samples from these ALS/PDC patients showed high contents of BMAA. More recently, unusually high incidents of ALS correlating with the occurrence of BMAA producing cyanobacteria have been found in New Hampshire^6^ and in southern France^7^. Further evidence supporting BMAA as a pathogenic factor is the increased occurrence of this neurotoxin in brain samples from North American ALS and Alzheimer’s disease patients^8^. Because BMAA has been shown to bioaccumulate within major food webs^9^, BMAA represents a potential environmental risk factor to human health.

The neurotoxic effect of BMAA exposure has been shown to be a result both from BMAA-induced overstimulation of glutamate responsive receptors^10^,^11^, and from misincorporation of BMAA into human neuroproteins^12^,^13^.

The β-carbamate of BMAA which forms in the presence of bicarbonate^14^ has been shown to act as an agonist to NMDA^10^,^11^, AMPA/kainate^15^ and mGluR5^16^ receptors. BMAA-induced activation of mGluR5 leads to decreased protein phosphatase 2A activity followed by tau hyperphosphorylation^17^. The latter is a phenomenon associated to pathologies of Alzheimer’s disease and Parkinsonism. Also, an increase in modified and aggregated TAR DNA-binding protein 43 (TDP-43), a hallmark of ALS, has been found to associate with BMAA exposure in both neuroblastoma cells^18^ and in injected and dietary exposed animals^19^,^20^. The changes in TDP-43 were observed together with increased levels and phosphorylation in glycogen synthase kinase 3 (GSK3) isoforms.

Misincorporation of BMAA into cellular proteins results in ER stress, redox imbalance, development of autofluorescent bodies and eventually caspase-dependent apoptotic cell death^12^,^21^,^22^. This misincorporation is shown to be protein synthesis dependent and the observed cellular effects of BMAA can be inhibited by L-serine^12^. Most possibly misincorporation of BMAA would affect not only protein folding and assembly, but also cell signaling regulated through serine phosphorylation.

Zebrafish has proven to be an excellent model for neurodegenerative diseases^23^,^24^. Basic structures of the zebrafish central nervous system are highly conserved compared to their disease-related human counterparts. Also most human disease genes are found as functional orthologues in zebrafish. The BMAA-targeted glutamate receptors are expressed from an early embryonic stage^25^,^26^. The rapid embryonal development and the emerging toolbox for genetic manipulation add to the value of using zebrafish as a model for humane diseases.

In the present study, we have characterized the effect of non-lethal exposure of early-life stage zebrafish to BMAA in respect to altered physiology and protein expression patterns.

## Results

### BMAA exposed embryos have reduced heart rate, but show no phenotypic changes

**Figure 1:**
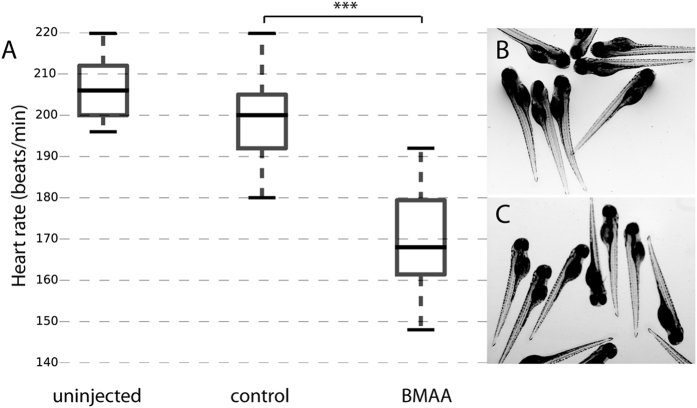
BMAA reduces heart rate, but does not alter larvae phenotype. Zebrafish embryos were injected at the one-cell stage with 4.2 nL (6.5 ng) of BMAA, equivalent volume of solvent control or left un-injected. (**A**) Box plot of heart rate (beats/min) measured at 3 dpf. Middle line in box represents the median, lower box bound the first quartile, and upper box bound the third quartile. n = 19–37 from two independent experiments, p < 0.001 (**B**) Un-injected larvae at 4 dpf. (**C**) BMAA-injected larvae at 4 dpf.

Zebrafish embryos were injected with 6.5–88 ng BMAA into the yolk sac at the one-cell stage. Embryos injected with 6.5 ng BMAA showed a significant reduced heart rate at 3 days post fertilization (dpf) compared to injected controls (Fig. 1A). No signs of pericardial oedema or other phenotypic changes of larvae injected with 6.5–88 ng BMAA were observed (Fig. 1B,C), data not shown). Neither did survival rate in BMAA-injected embryos differ from control-injected animals (data not shown).

### BMAA increased the levels of glycogen synthase kinase 3 α and β (GSK3 α/β)

**Figure 2:**
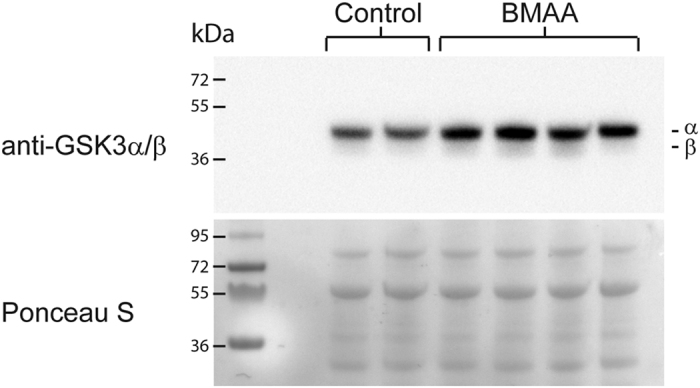
Embryonal BMAA-injection increases GSK3α/β expression in larvae. Embryos injected with 6.5 ng BMAA or solvent control at one-cell stage were harvested 4 dpf. Proteins were extracted from whole larvae (n > 50) and subjected to Western blotting using anti-GSK-3α/β. Ponceau-S staining was used as loading control. Blot shows control and BMAA-injected samples from two individual experiments.

Since the BMAA-injected larvae showed no signs of phenotypic abnormalities compared to what was observed by Purdie *et al*.^27^, we also determined whether the BMAA-injected larvae showed an induction of GSK3 isoforms. Previous studies in neuroblastoma cells and cerebellum of BMAA-exposed rats have shown that the levels of both α and β isoforms of GSK3 increase as a response to BMAA^28^,^29^. We analyzed GSK3 α/β levels in total lysates of BMAA (6.5 ng) and control injected 4 dpf larvae using Western blotting. As can be seen in Fig. 2 BMAA-injected embryos showed increased levels of both α and β isoforms of GSK3 at 4 dpf. Larvae (4 dpf) injected with 6.5 ng BMAA were therefore selected for further label-free proteomics analysis.

### Label-free proteomics analysis

**Figure 3:**
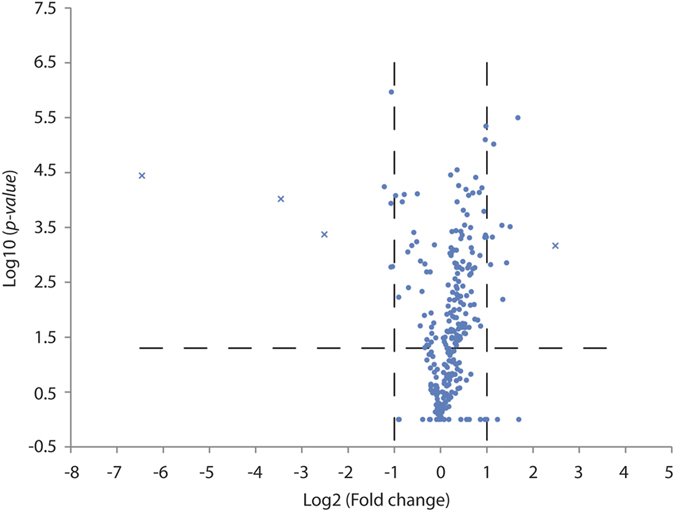
Volcano plot of BMAA-induced protein changes observed by label-free quantitation. The protein expression ratio of BMAA/Control (log_2_ scale) in label-free quantitation was plotted against the −log_10_ of the probability calculated by *t*-test. Proteins with missing values are replaced by random numbers drawn from the normal distribution, and are marked as x in the graph. The dashed lines represent the applied threshold values (*p*-value ≤ 0.05, Fold change ≥ 2).

Protein extracts from total larvae were analyzed using a label-free proteomics approach to identify proteins that were differentially regulated in BMAA-exposed larvae compared to controls. We searched both towards the reviewed *Danio rerio* database with 2968 entries and the combined UniProtKB (reviewed & unreviewed) database with 58693 entries. In total, 480 (reviewed) and 2833 (UniProtKB) proteins were identified and quantified in the label-free approach. 295 (reviewed) and 1960 (UniProtKB) proteins meet the criteria (see material and methods section) for further statistical analysis to compare BMAA-exposed to control-injected larvae (Supplementary Tables S1–4). An overview of the number of proteins and peptides identified in each technical replicate can be seen in Supplementary Table S5. To limit the possibilities of focusing on false positive protein identifications we choose to work further on the dataset obtained with the curated database. Volcano plot in Fig. 3 shows the log2-fold change and p-value of all proteins in BMAA-exposed larvae compared with controls identified using the curated database. Of these, 17 proteins were found to be regulated 2-fold or more (Fig. 3 and Table 1). This included four proteins that were either found only in BMAA-exposed or only in controls. Imported values reflecting the lower detection limit of the mass spectrometry analysis were imported for proteins that were detected only in one condition in order to reflect fold regulation in Fig. 3. A multi-scatter plot showing the high reproducibility of the technical repeats can be seen in Supplementary Figure 1.

### Proteins regulated by BMAA

A list of differentially regulated proteins according to their BMAA/control ratio is presented in Table 1. Eight proteins were shown to be more than two-fold up-regulated in BMAA-exposed animals, and one protein was observed only after BMAA exposure. Five proteins were down-regulated in BMAA-treated animals and three proteins were only observed in controls.

The proteins that were influenced by BMAA treatment have been shown to be associated to glutamate signaling, protein homeostasis, ROS response and autophagy/cell death^30^,^31^,^32^,^33^,^34^,^35^,^36^,^37^,^38^,^39^,^40^,^41^.

As BMAA appears to act as a stimulator of glutamate responsive receptors, we would expect to observe regulation of proteins associated with glutamate signaling. Indeed, seven out of the 17 regulated proteins could be associated with glutamate signaling. These included the up-regulated AP-2 complex subunits muA/B (AP-2)^30^, Fatty-acid amide hydrolase 2B (FAAH2b)^31^ and Ca^2+^/calmodulin-dependent kinase II α/δ (CaMKIIα/δ)^32^. Additionally, Glyceraldehyde-3-phosphate dehydrogenase (GAPDH) and Glyceraldehyde-3-phosphate dehydrogenase-2 (GAPDH-2), which were found to be down-regulated in BMAA-exposed animals, could also be associated to glutamate signaling. While GAPDH has an essential role in glycolysis, it has also been shown to bind to the AMPA glutamate receptor^33^. Protein RCC2 homolog (RCC2) may indirectly regulate glutamate signaling, as it has been shown to be a dual regulator of RAC1 and ARF6^34^ which are involved in glutamate receptor recycling^35^. Another BMAA-regulated protein associated to the synaptic endosomal system is the PP2A inhibitor Acidic leucine-rich nuclear phosphoprotein 32E (ANP32e)^36^.

BMAA has been shown to lead to ER stress as a result of mis incorporation of BMAA into cellular proteins. Two BMAA-regulated proteins: Protein transport protein Sec61 subunit alpha-like 1 (SEC61a) and RuvB-like 2 (RUVBL2), have been shown to be involved in ER stress regulation^37^.

Amongst the upregulated proteins were proteins involved in protein biosynthesis and RNA processing: Eukaryotic translation initiation factor 3 subunit A and C (eIF3a/c) and Cleavage and polyadenylation specificity factor 5 (CPSF5). Two other protein biosynthesis associated proteins: 40S ribosomal protein S21 (RPS21) and Phenylalanine-tRNA ligase α (FARSA) were downregulated in BMAA. Down-regulation of RPS21 has also been observed as a response to oxidative stress^38^.

The most up-regulated protein is Serrate RNA effector molecule homolog (ARS2), a transcription factor postulated to be a protector of ROS-induced cell toxicity^39^.

Lastly, two more proteins were shown to be down-regulated in BMAA-exposed animals. These were Epididymal secretory protein E1 (NPC2) which is linked to lysosomal disorder and autophagy^40^, and oxygen carrier Hemoglobin subunit beta (BA1). Decrease in mitochondrial hemoglobin is observed during developmental-related neuronal loss and in Parkinson’s Disease (PD)^41^.

### Verification of mass spectrometry data

**Figure 4:**
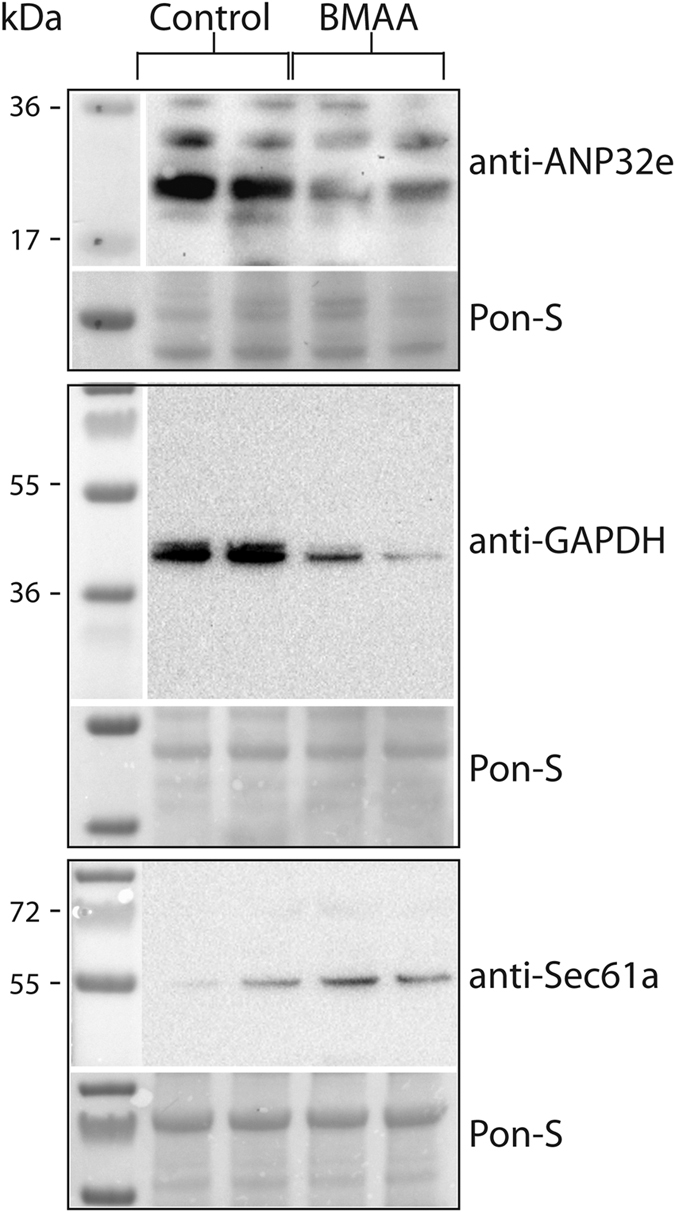
Verification of mass spectrometry results. Total protein extracts (40 μg per lane) from BMAA- and control-injected larvae were separated by SDS-PAGE and transferred to PVDF membranes for Western blotting with the indicated antibodies. Ponceau-S staining was used as loading control. Blots show control and BMAA-injected samples from two individual experiments.

The possibility to verify the mass spectrometry data (Table 1) by Western blotting is somewhat restricted due the limited availability of zebrafish recognizing antibodies. Candidates were therefore chosen based on the number of covering tryptic peptides showing similar regulation and homology to proteins with available targeting antibodies. Western blot towards using anti-ANP32e showed two bands that both were down-regulated in BMAA-exposed animals, thus verifying the mass spectrometry data. Zebrafish ANP32e is predicted to be 28 kDa and is most likely the high intensity band. ANP32a which is highly homologues to ANP32e has a slight higher Mw (Fig. 4). Also, the BMAA-induced down-regulation in GAPDH and the Sec61a up-regulation were verified by Western blotting (Fig. 4).

**Figure 5:**
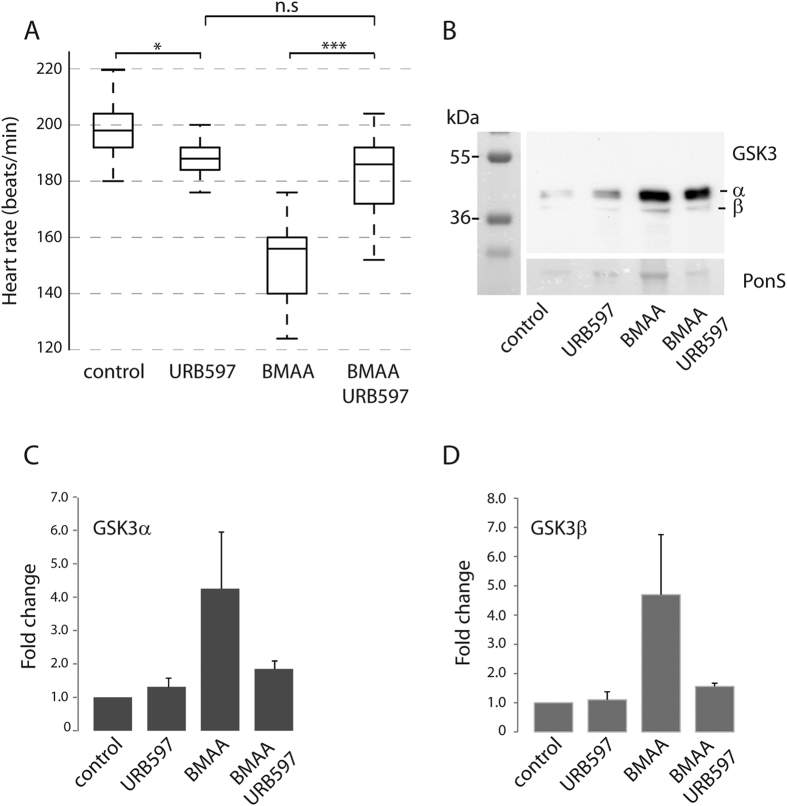
FAAH inhibitor URB597 reduces the effect of BMAA. (**A**)FAAH inhibitor URB597 reduces the effect of BMAA on the heart rate (beats/min). Embryos were injected with BMAA or BMAA+URB597 at the one-cell stage. Heart rate was monitored at 3 dpf. Middle line in box represents the median, lower box bound the first quartile, and upper box bound the third quartile. n = 30–40 from three individual experiments, p < 0.001. (**B**) GSK3α/β expression in total lysates from injected larvae at 4 dpf. Ponceau-S staining was used as loading control. (**C**) GSK3α abundance. (**D**) GSK3β abundance. Data in C and D are expressed fold change relative to Ponceau-S expression in which bicarbonate-injected controls were used as the reference. Data are expressed as means +/− SEM from three independent experiments.

Our label free proteomics analysis showed FAAH2b to be up-regulated in BMAA-exposed animals (Table 1). Knock-out of FAAH in a mice model of ALS has been shown to inhibit the progression of neurodegeneration. It was therefore tempting to test if the FAAH inhibitor URB597 could inhibit BMAA-induced reduction in heart rate (Fig. 1) and increased GSK3 expression (Fig. 2). Indeed, URB597 protected against BMAA-induced reduction in heart rate (Fig. 5A). Western blots also showed a tendency, although not significant, of URB597 to inhibit BMAA-induced GSK3α and β increase (Fig. 5B–D).

## Discussion

Recently, Cox *et al*. showed that dietary exposure of vervets to BMAA led to neurofibrillary tangles and amyloid deposits^20^. These are diagnostic hallmarks of several neurodegenerative diseases and similar histopathological findings as described in ALS/PDC patients of Guam^42^. The mechanisms leading these histopathological changes and neuronal loss are far from understood. BMAA-exposed animals and cells also show aberrant glutamate signaling, redox imbalance, and ER stress^16^,^21^, which all have been suggested to be involved in neurodegenerative diseases, although the causal effects leading to this are unclear. In this study we have conducted a label free proteomics approach to further characterize the mechanism of BMAA-induced toxicity. Zebrafish have proven to be an excellent model for neurodegenerative diseases and was chosen as the *in vivo* model.

In contrast to what was observed in an earlier study of zebrafish exposed to BMAA^27^, we did not observed any BMAA-induced malformations or cardiac oedema in our studies with sub-lethal exposure (Fig. 1). But, the BMAA-injected dosage was sufficient to significantly decrease heart rate and also to up-regulate GSK3 α and β expression, supporting previous studies of BMAA-exposed cell culture and BMAA-injected rats^27^,^28^.

In our label free proteomics study, we identified 480 proteins in the total extracts of 4 dpf larvae using a reviewed *Danio rerio* database containing 2968 proteins. Of the 480 identified proteins only 17 proteins were significantly >2 fold regulated in the BMAA-exposed zebrafish larvae compared to controls (Table 1). All these BMAA-regulated proteins were proteins known to have a regulatory function in glutamate receptor activity/recycling, ER stress, protein biosynthesis, ROS, autophagy or neuronal cell death, thereby giving further insight into cell signaling pathways leading to previously observed BMAA-induced cell toxicity.

The BMAA-regulated CaMKIIα/β, FAAH, GADPH are known to associate to and regulate glutamate receptor activity. CaMKII enzyme is composed of multiple subunits (α, β, γ, δ) either as holo- or heteroenzymes. The BMAA-induced regulation of CaMKII was based on intensity of unique peptides found in both α and δ isoforms. CaMKIIα is the isoform predominantly expressed in neuronal tissue where it can regulate glutamate receptor localization and function by direct phosphorylation^32^. On the other hand, CaMKII is also recognized as an oxidative sensor^43^ and a key player in cell death induced by phosphatase inhibiting cyanobacterial toxins^44^. Glutamate-induced excitotoxicity has previously been shown to up-regulate CaMKII^45^. The enzyme fatty acid amide hydrolase (FAAH) selectively degrades arachidonoyl ethanolamide, AEA, an intercellular messenger involved in synaptic retrograde signaling through inhibiting release of neurotransmitters as glutamate^31^. The increase in FAAH as observed in BMAA-exposed zebrafish larvae (Table 1) may therefore facilitate the effect of BMAA as a glutamate agonist. We therefor targeted FAAH using URB597 (Fig. 5) to see whether this could inhibit the effect of BMAA. Indeed, URB597 inhibited the BMAA-induced reduction in heart rate (Fig. 5A). URB597 also seemed to affect the BMAA-induced increase in GSK3 levels (Fig. 5B-D), thus suggesting that BMAA-associated increase in GSK3 levels at least in some part is regulated through BMAAs receptor agonist action. Interestingly, FAAH has been proposed as a therapeutic target for both ALS and PD^46^,^47^. GADPH is considered as a canonical enzyme in glycolysis, but it is also known to be an extracellularly binding partner of glutamate receptors^48^ and is also known to mediate ROS-induced neuronal cell death^49^. The excitotoxic effect of BMAA was further supported by the observed regulatory effect on AP-2, RCC2, ANP32e, proteins associated to glutamate receptor endocytosis and recycling. AP-2 functions together with clathrin to regulate glutamate receptor abundance and endocytosis^30^. As already mentioned, RCC2 and ANP32e, are also known to be associated to the synaptic endosomal system^33^,^34^.

BMAA incorporation into proteins has been found to induce ER stress in human neuroblastoma cells^21^. ER stress activates a signaling cascade called Unfolded Protein Response (UPR). BMAA-induced ER stress in zebrafish was reflected in an up-regulation of Sec61, a pore protein of the ER (Table 1). SEC61 establishes a protein pore in the ER, in which newly made proteins can enter the ER. SEC61 is induced by ER stress in an UPR-dependent manner^50^. Additionally, supporting BMAA-induced ER stress is the down-regulation of RUVBL2 (Table 1). Degradation of RUVBL2 has been shown to induce ER stress by up-regulating the transcript of a specific set of UPR-related genes^37^. Meanwhile, the UPR-response enhances degradation of misfolded proteins stress cell response to maintain protein homeostasis can also be directed towards protein synthesis at all levels. Our data reflects this as several of the BMAA-regulated proteins are known to be involved in protein synthesis and RNA processing; eIF3a/c, CPSF5, RPS21, and FARSA. Interestingly, FARSA was recently shown to be inverse correlated with *C9orf72*^51^. Mutations in the *C9orf72* are the most common mutation associated to ALS.

In conclusion, we have identified novel actors in both BMAA-induced excitotoxic and metabolic disturbing cell signaling. The study also shows that the endocannabinoid system, through FAAH, is activated by BMAA. We have opened for zebrafish to be a highly useful translational model in the study of the relation between BMAA and neurodegenerative disease, and further studies can benefit from the large molecular toolbox offered in zebrafish.

## Material and methods

### Materials

L-BMAA, URB597, anti-ANP32e (SAB2100124), iodoacetamide, amoniumbicarbonate and urea were purchased from Sigma Aldrich. Anti-GSK-3α/β (sc-7291) and anti-GAPDH (sc-32233) and anti-Sec61a (sc-12322) were from Santa Cruz. Sequence grade trypsin was from Promega. Acetonitrile (ACN), water and trifluoretic acid (TFA) were all HPLC grade and purchased from Sigma Aldrich.

### Animal maintenance

Tübingen AB (TAB) zebrafish were housed and experiments carried out at the zebrafish facility at the Department of Molecular Biology, University of Bergen. The facility is run in agreement with European Convention for Protection of Vertebrate Animals used for Experimental and Other Scientific Purposes. No ethics committee approval was required for experiments since they were all carried out on animals no older than 5 dpf. Adult zebrafish were maintained at 26–28 °C on a 14/10 light/dark cycle and fed twice daily. Embryos were obtained by natural mating and raised in E3 buffer (5 mM NaCl, 0.17 mM KCl, 0.33 mM MgSO_4_) at 28 °C.

### Exposure to BMAA, FAAH inhibitor and heart rate analysis

BMAA was diluted in 10 mM NaHCO_3_ to obtain a 65 mM solution. Embryos were injected with 4.2 nL (6.5 ng) BMAA solution into the yolk sac at one cell stage. Control embryos were injected with bicarbonate solution. Control injections were performed on eggs from the same mating batch as those that were BMAA-injected. FAAH inhibitor (20 nM) was co-injected with BMAA or control solution. Embryos and larvae were observed using a light microscope every day before sampling for protein extraction at 4 dpf.

Heart rates were determined by counting the number of atrial contraction in 15 seconds in embryos at 3 dpf using a Zeiss Stereozoom light microscope.

### Protein extraction

Larvae 4 dpf were washed twice in PBS and homogenized by sonication (2 × 15 sec) in 10 mM K_2_HPO4, 10 mM KH_2_PO_4_, 1 mM EDTA, 0.6% CHAPS, 0.2 mM Na_3_VO_4_, 50 mM NaF with protease cocktail (Roche). Five μl homogenization buffer was added per embryo. Samples were pelleted at 16 000 g for 15 min and supernatant was either used directly or stored at −80 °C.

### Western blotting

Protein extracts (40 μg/well) were separated by SDS-PAGE and transferred to PDVF membranes. Ponceau S staining was used as loading control. Western blotting was performed with anti-GSK-3α/β (1:500 1 hr at RT) anti-GAPDH (1:500 1 hr at RT), anti-ANP32e (1:1000 1 hr at RT) or anti-Sec61a (1:500 1 hr at RT) followed by peroxidase-conjugated anti-mouse antibody (715-035-150, Jackson Immuno Research laboratories) or anti-goat antibody (711-035-152, Jackson Immuno Research laboratories).

### Sample preparation for mass spectrometry

Protein extract 30 μg was denaturated with 200 μl 8 M urea on a Microcon YM-30 (Millipore, Cat. MRCF0R030) according to Filter Aided Sample Preparation (FASP) method described by Wisniewski *et al*.^52^. For reduction, 100 μl 10 mM DTT was added and incubated for 1 hr in room temperature followed alkylation with 100 μl 0.05 M iodoacetamide for 20 min in room temperature. Trypsin (enzyme:protein ration 1:100) (Promega) was added and the proteins were digested over night at 37 °C. After digestion 40 μl of 0.05 M ABC was added and samples were spun down before 50 μl 0.5 M NaCl was added. The samples were acidified with 10% TFA until a final concentration of 1% TFA. Peptides were up-concentrated and desalted on C18 Oasis™ μElution plates (Waters, Milford, MA), the sample volume were reduced by speed vac.

### Label-free mass spectrometry analysis

About 0.5 μg protein as tryptic peptides dissolved in 5% ACN, 0.1% TFA, were injected into an Ultimate 3000 RSLC system (Thermo Scientific, Sunnyvale, California, USA) connected online to a Q-Excative HF mass spectrometer (Thermo Scientific, Bremen, Germany) equipped with EASY-spray nano-electrospray ion source (Thermo Scientific). Each sample was injected three times.

The sample was loaded and desalted on a pre-column (Acclaim PepMap 100, 2 cm × 75 μm i.d. nanoViper column packed with 3 μm C18 beads) at a flow rate of 5 μl/min for 5 min with 0.1% TFA.

Peptides were separated during a biphasic ACN gradient from two nanoflow UPLC pumps (200 nl/min) on a 50 cm analytical column (PepMap RSLC, 50 cm × 75 μm i.d. EASY-spray column, packed with 2 μm C18 beads). Solvent A and B were 0.1% TFA (vol/vol) in water and 100% ACN respectively. The gradient composition was 5%B during trapping (5 min) followed by 5–8%B over 0.5 min, 8–35%B for the next 134.5 min, and 35–90%B over 15 min. Elution of very hydrophobic peptides and conditioning of the column were performed during 15 minutes isocratic elution with 90%B and 20 minutes isocratic conditioning with 5%B. The total length of the LC run was 195 min.

The eluting peptides from the LC-column were ionized in the electrospray and analyzed by the Q-Excative HF. The mass spectrometer was operated in the DDA-mode (data-dependent-acquisition) to automatically switch between full scan MS and MS/MS acquisition. Instrument control was through Q Excative HF Tune 2.4 and Xcalibur 3.0.

MS spectra were acquired in the scan range 375–1500 m/z with resolution R = 120 000 at m/z 200, automatic gain control (AGC) target of 3e6 and a maximum injection time (IT) of 100 ms. The 12 most intense eluting peptides above intensity threshold 2e4, and charge states 2 or higher, were sequentially isolated to a target AGC value of 1e5, resolution R = 30 000, IT of 110 ms and normalized collision energy of 28%. The isolation window was set to 1.6 m/z with an isolation offset of 0.3 m/z and a dynamic exclusion of 25 seconds. Lock-mass internal calibration (m/z 445.12003) was used. Three technical replicates were performed.

### Data interpretation and statistics

The raw files were searched in MaxQuant (version 1.5.3.28) against a *Danio rerio*, a reviewed database from UniProt/SwissProt (downloaded October 2015, 2945 entries), and the combined UniProtKB database (downloaded May 13 2016, 58693 entries). Following search parameters were used; carbamidomethyl (Cys) as fixed modification, oxidation (Met), phosphorylation (STY) and acetyl (Protein N-terminal) as variable modifications. We allowed for two miss cleavages of trypsin, 20 ppm for precursors and 0.6 Da for fragment ion mass tolerance, and false discovery rate (FDR) at 1%. Only unmodified and two unique + razor peptides were used for quantifications. Perseus (1.5.2.6) was used for further analysis. Proteins identified by site, reverse hit and potential contaminants were removed. The protein list was further reduced by only keeping proteins with at least 3 out of 6 valid values. The LFQ intensity values were log2 transformed, and proteins were considered significant if they passed the *two sample t-test*, with the following settings; S0 = 2 and FDR 0.01. P-values were given as Log10 value and the difference between BMAA and control was given by log2 difference in Perseus. Proteins that fulfilled the above criteria, but had missing numbers were quantitated (BMAA/Control) by replacing missing values by random numbers drawn from the normal distribution.

## Additional Information

**How to cite this article**: Frøyset, A. K. *et al*. Quantitative proteomics analysis of zebrafish exposed to sub-lethal dosages of β-methyl-amino-L-alanine (BMAA). *Sci. Rep.* **6**, 29631; doi: 10.1038/srep29631 (2016).

## Supplementary Material

Supplementary Information

Supplementary Dataset S1

Supplementary Dataset S2

Supplementary Dataset S3

Supplementary Dataset S4

## Figures and Tables

**Table 1:** Proteins regulated by BMAA.

Acc no	Gene name	Fold* regulation	Protein name	Unique peptides	Score	p-value	Known function relation to				Keyword (UniProtKB)
							Glutamate receptor	Protein homeost.	ROS	Autophagy Cell death	Biological process
**Upregulated proteins**											
Q66I22	srrt	3.19	Serrate RNA effector molecule homolog	5	37.8	3.20E-06			x		RNA-mediated silencing
Q05AM4	faah2b	2.83	Fatty-acid amide hydrolase 2-B	3	12.8	0.0003	x				hydrolase activity
Q6DEH3	camk2α/δ	2.69	Ca2+/calmodulin-dependent protein kinase type II α/δ	2	16.9	0.0014	x		x		ser/thr-protein kinase
Q6NYE2	rcc2	2.54	Protein RCC2 homolog	5	59.8	0.0065	x				cell cycle
Q6PCR7	eif3a	2.5	Eukaryotic translation initiation factor 3 subunit A	9	66.6	0.0003		x			protein biosynthesis
Q6PFQ2	eif3c	2.21	Eukaryotic translation initiation factor 3 subunit C	10	323.3	9.61E-06		x			protein biosynthesis
Q90ZM2	sec61a	2.17	Protein transport protein Sec61 subunit alpha-like 1	4	4.9	5.08E-05		x			protein transport
Q7T3C6	cpsf5	2.11	Cleavage and polyadenylation specificity factor 5	2	61.9	0.0005		x			mRNA processing
Q7ZW98	ap2m1a/b	n.d	AP-2 complex subunit mu	3	24.6		x				endocytosis, protein transport
**Downregulated proteins**											
Q6NUW5	anp32e	0.43	Acidic leucine-rich nuclear phosphoprotein 32 E	6	131.2	5.72E-05	x				chaperone, phosphatase inhibitor
Q1JPX3	farsa	0.47	Phenylalanine–tRNA ligase alpha subunit	4	172.6	0.0017		x			protein biosynthesis
Q5MJ86	gapdh-2	0.47	Glyceraldehyde-3-phosphate dehydrogenase 2	14	323.3	0.0001	x		x		glycolysis
Q5XJ10	gapdh	0.48	Glyceraldehyde-3-phosphate dehydrogenase	14	323.3	1.07E-06	x		x	x	apoptosis, glycolysis
Q9DGJ3	npc2	0.49	Epididymal secretory protein E1	4	58.5	0.0016				x	lipid metabolism
P83571	ruvbl2	n.d.	RuvB-like 2	3	51.3			x			transcription regulation
Q7ZUG5	rps21	n.d.	40S ribosomal protein S21	3	8.9			x	x		translation
Q90486	ba1;ba2	n.d.	Hemoglobin subunit beta	2	16.4					x	oxygen transport, transport

Fold regulation shows BMAA/control. Proteins only detected in triplicates of either BMAA or control have fold regulation not detemined (n.d.).
