# Supplementary material for: Quantitative proteomics analysis of zebrafish exposed to sub-lethal dosages of β-methyl-amino-L-alanine (BMAA)
Source: Sci Rep. 2016 Jul 12;6:29631. doi: 10.1038/srep29631 (PMC4940735; doi:10.1038/srep29631)
Supplement: Supplementary Information [file srep29631-s1.pdf]

Quantitative proteomics analysis of zebrafish exposed to sub-lethal dosages of β-methyl-amino-L-alanine (BMAA)

Ann Kristin Frøyset, Essa Ahsan Khan, and Kari Espolin Fladmark

Department of Molecular Biology, University of Bergen, Thormøhlensgate 55, 5020 Bergen, Norway.

Legend supplementary data

Quantitative proteomics analysis of zebrafish exposed to sub-lethal dosages of β-methyl-amino-L-alanine (BMAA)

Ann Kristin Frøyset, Essa Ahsan Khan, and Kari Espolin Fladmark

Department of Molecular Biology, University of Bergen, Thormøhlensgate 55, 5020 Bergen, Norway.

Figure S1: Multi-scatter correlation plots of technical replicates. The LFQ intensities of each replicate of BMAA and CTR samples are plotted against each other. Pearson correlations are displayed for individual graph, showing a high level of reproducibility for all replicate and between BMAA and CTR samples.

Table S1: Identified proteins using the reviewed UniProt database with 2945 entries. For search criteria and data filtration see Material and Methods section.

Table S2: Peptides identified using the reviewed UniProt database with 2945 entries. For search criteria see Materials and Methods section. Reverse and potential contaminants are removed.

Table S3: Identified proteins using the combined (unreviewed & reviewed) UniProtKB database with 58693 entries. For search criteria and data filtration see Material and Methods section.

Table S4: **Peptides identified using the combined (unreviewed & reviewed) UniProtKB database with 58693 entries.** For search criteria see Materials and Methods section. Reverse and potential contaminants are removed.

Table S5: Overview of total proteins and peptides identified in each technical replicate using reviewed UniProt database and combined (unreviewed & reviewed) UniProtKB database.

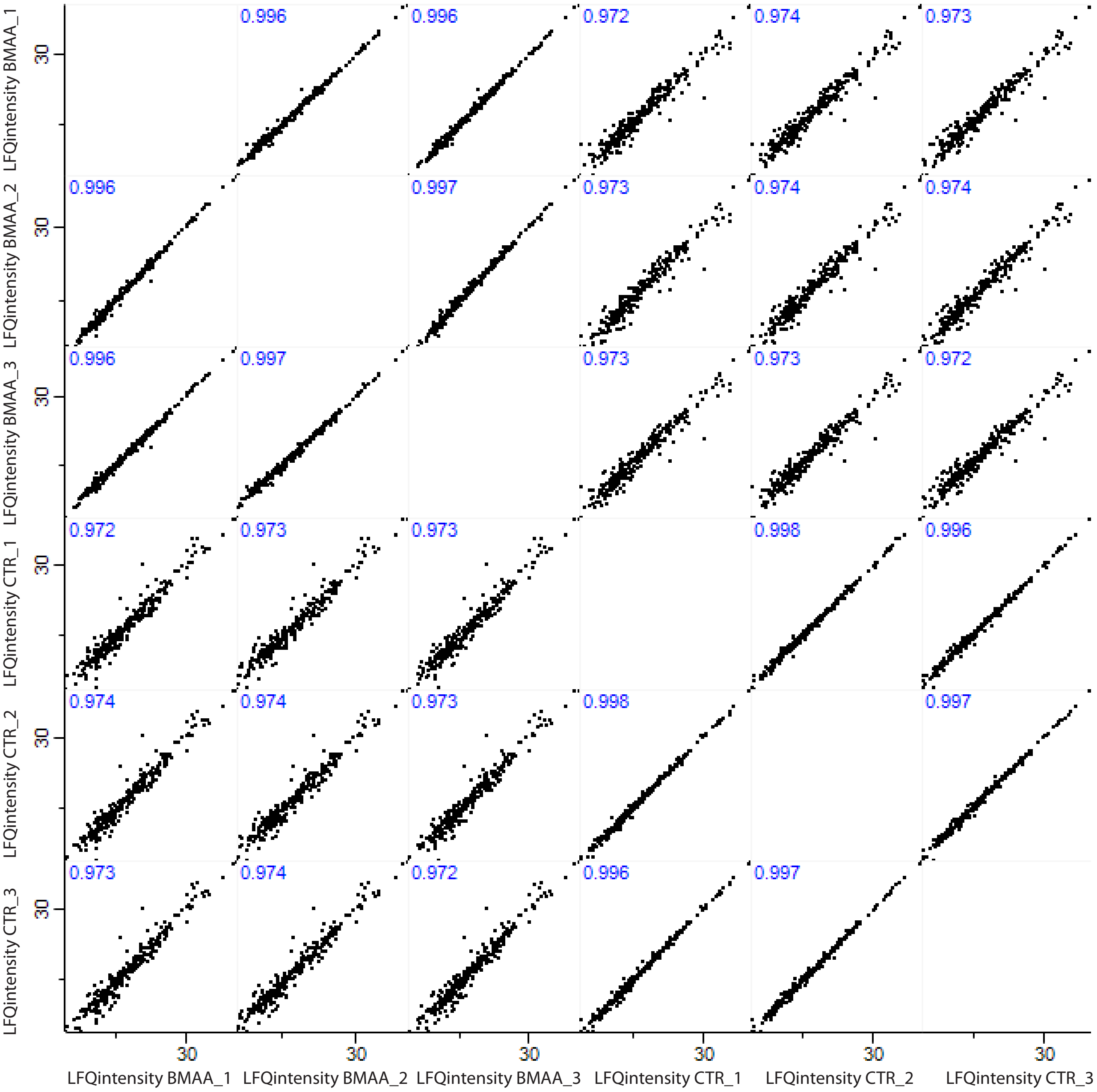
0.996
0.996
0.972
0.974
0.973
0.996
0.997
0.973
0.974
0.974
0.996
0.997
0.973
0.973
0.972
0.972
0.973
0.973
0.998
0.996
0.974
0.974
0.973
0.998
0.997
0.973
0.974
0.972
0.996
0.997
30
LFQintensity BMAA_1
LFQintensity BMAA_2
LFQintensity BMAA_3
LFQintensity CTR_1
LFQintensity CTR_2
LFQintensity CTR_3

Quantitative proteomics analysis of zebrafish exposed to sub-lethal dosages of β-methyl-amino-L-alanine (BMAA)

Ann Kristin Frøyset, Essa Ahsan Khan, and Kari Espolin Fladmark
Department of Molecular Biology, University of Bergen, Thormøhlensgate 55, 5020 Bergen, Norway.

	**Number of peptides**		**Number of proteins**	
	**UniProtKB**	**Reviewed**	**UniProtKB**	**Reviewed**
**CTR_1**	14653	2028	1879	285
**CTR_2**	14660	2029	1881	281
**CTR_3**	14620	2014	1900	284
**BMAA_1**	13441	1870	1827	270
**BMAA_2**	13512	1868	1834	274
**BMAA_3**	13489	1861	1829	272

Suppl. Table 5 Total number of peptides and proteins identified in replicates. Table shows peptides and proteins identified using combined database UniProtKB) and reviewed database meeting the criteria set for Suppl. Tables 1 and 2.
